# Diminished Expression of Complement Regulatory Proteins on Peripheral Blood Cells from Systemic Lupus Erythematosus Patients

**DOI:** 10.1155/2012/725684

**Published:** 2012-06-12

**Authors:** Ana Paula Alegretti, Laiana Schneider, Amanda Kirchner Piccoli, Odirlei Andre Monticielo, Priscila Schmidt Lora, João Carlos Tavares Brenol, Ricardo Machado Xavier

**Affiliations:** ^1^Serviço de Patologia Clínica, Hospital de Clínicas de Porto Alegre, Universidade Federal do Rio Grande do Sul, Rua Ramiro Barcelos, 2350, 2° Andar, 90035-903 Porto Alegre, RS, Brazil; ^2^Serviço de Reumatologia, Hospital de Clínicas de Porto Alegre, Universidade Federal do Rio Grande do Sul, Rua Ramiro Barcelos, 2350, 6° Andar, 90035-903 Porto Alegre, RS, Brazil

## Abstract

CD55, CD59, CD46, and CD35 are proteins with complement regulatory (Creg) properties that ensure cell and tissue integrity when this system is activated. The aim of this study was to evaluate the Creg expression on peripheral blood cells from SLE patients and its association with cytopenia and disease activity. Flow cytometric analyses were performed on blood cells from 100 SLE patients and 61 healthy controls. Compared with healthy controls, we observed in SLE patients with lymphopenia and neutropenia decreased expression of CD55, CD59, and CD46 (*P* < 0.05). In SLE patients with anemia, CD59 and CD35 were decreased on red blood cells. Furthermore, there was a negative correlation between CD55 and CD59 on neutrophils and the disease activity. The results suggest there is an altered pattern of Creg expression on the peripheral blood cells of SLE patients, and the expression is correlated with disease activity and/or with activation of the complement system.

## 1. Introduction

The complement system (CS) represents the first defense line of innate immunity; it acts facilitating the phagocytosis of immune complexes, pathogens, and apoptotic cells and forming the membrane attack complex (MAC), resulting in cell lysis. This powerful defense system is composed of multiple components (>60 different proteins and activation products) that trigger in a cascade-type system [1].

The complement as a central defense system is immediately activated within seconds upon entry of a pathogen into the human host through three pathways: the classical (triggered by antibody-antigen complexes), the lectin (triggered by carbohydrates on the surface of bacteria), and the alternative pathways (spontaneous and continuous process which is initiated by the conformational change of C3). These three pathways use different proteins to produce C3 and C5 convertases, which involve cleavage of C2 and C4 (classical and lectin pathway) or the cleavage of factor B by factor D (alternative pathway). All result in the formation of the lytic MAC (membrane-attack complex: C5b-9) [2, 3]. Activation of the complement system is a powerful drive to initiate inflammation but can, if unregulated, lead to severe tissue damage and disease. Based on their potent damaging capacity, the generation and targeting of complement effector compounds are tightly regulated [4].

Normal cell membranes express complement regulatory (Creg) proteins that regulate activation of the complement system and provide essential protection against self-damage [5]. There are four major human cell surface Creg proteins: CD59 (membrane inhibitor of reactive lysis—MIRL), which is a complement membrane inhibitor that blocks assembly of the MAC by binding to C8 and C9 [6], CD55 (decay accelerating factor—DAF), which accelerates the disassembly of preformed C3 and C5 convertases [7], CD46 (membrane cofactor protein), which acts as a cofactor for the factor-I-mediated cleavage of the activated complement components C3b/C4b [8], and CD35 (complement receptor type I, CR1), which is also involved in the regulation of C3 fragment deposition and serves as a cofactor for the degradation of C3b by factor I [4]. These Creg proteins are present on the cell surface of whole blood cells, except the CD46, which is not expressed on RBCs. It has been reported that the production and the expression of some of these complement regulatory proteins are altered in autoimmune diseases and that inherited deficiencies of the complement system components are associated with a high prevalence of systemic lupus erythematosus (SLE), glomerulonephritis, and vasculitis [9–11].

The complement system is integrally involved in the pathogenesis of tissue injury in SLE. Tissue deposition of immunoglobulin is a characteristic feature of SLE and can cause continued complement activation by the classical pathway [10]. Therefore, potential differences on the expression of the Creg proteins could implicate different susceptibilities to complement-mediated damage and be clinically significant. Particularly, altered expression on blood cells could be related to cytopenic changes common in this disease. Earlier studies have shown that expression of CD35 [12–16], CD55, and CD59 [17, 18] on erythrocytes and CD55 and CD59 [19–21] on lymphocytes are decreased in patients with SLE, but some of these findings were controversial. The current study aimed to evaluate the expression of CD55, CD59, CD46, and CD35 expression on peripheral blood cells from SLE and healthy controls using flow cytometry and its correlation with cytopenias on these patients.

## 2. Material and Methods

### 2.1. Subjects

One hundred patients that fulfilled the American College of Rheumatology classification criteria [22] for SLE and 61 healthy controls with no history of autoimmune diseases were included in the present study. SLEDAI (SLE disease activity index) [23] and SLICC (systemic lupus international collaborating clinics) damage index [24] were applied to each patient as a measurement of disease activity and cumulative damage, respectively.

SLE patients were followed up at the Rheumatology Clinic of Hospital de Clínicas de Porto Alegre, Brazil. The exclusion criterion was concomitant presence of overlap with another autoimmune disease. Peripheral blood samples were collected in Na-EDTA Vacutainer tubes. All SLE patients were receiving an immunosuppressive drug at the time of blood collection (mycophenolate mofetil, cyclophosphamide, azathioprine, methotrexate, cyclosporine, and/or rituximab). 

This study was performed with approval of the ethics committee of the Hospital de Clínicas de Porto Alegre, and all subjects were informed about the objectives and procedures of the study and gave their written informed consent.

### 2.2. Flow Cytometric Analysis of CD55, CD59, CD35, and CD46 on the Cell Membrane

For red blood cell (RBC) staining, 100 uL of diluted blood (with an optimal dilution with phosphate-buffered saline (PBS) to achieve 10000 RBC/uL) as placed into polystyrene tubes (Becton Dickinson (BD) Biosciences, San Diego, CA, USA) and as subjected to two-colour staining with 8 uL/test of fluorochrome-conjugated monoclonal antibodies (MoAbs) against CD55PE, CD59FITC, CD35PE, and CD46FITC (BD Biosciences, San Diego, CA, USA). After 20 min incubation at room temperature, samples were resuspended in 0.5 mL of PBS and cells were analysed on the flow cytometer.

For leukocyte staining, 100 uL of whole blood (with an optimal dilution to achieve 5000 cells/uL) as placed into polystyrene tubes and as subjected to two-colour staining with 8 uL of each antibody of fluorochrome-conjugated MoAbs against CD55PE, CD59FITC, CD35PE and CD46FITC (BD Biosciences, San Diego, CA, USA). After 15 min incubation at room temperature, 1.0 mL of FACSlyse (BD Biosciences, San Diego, CA, USA) was added and lysis was allowed for 10 min at room temperature. Samples were washed once and resuspended in 0.5 mL of PBS. 

50000 events were acquired and analysed on a FACSCalibur flow cytometer using CellQuest software (BD Biosciences, San Diego, CA, USA). Membrane intensity of CD55, CD59, CD46, and CD35, which is proportional to the number of CD55, CD59, CD46 and CD35 epitopes on the membrane, was estimated in the gated subpopulations by one-parameter histograms, and the relative mean fluorescence intensity (MFI) was recorded. The definition of positive and negative cells was set when staining with isotype control was performed, in order to set the gates and distinguish positive staining from autofluorescence and nonspecific antibody binding.

### 2.3. Serological Studies

Measurement of complement 3 (C3) and complement 4 (C4) is used to determine whether primary deficiencies or activation-related consumption of the complement components is present in SLE patients. C3 and C4 measurements were performed using the ADVIA 1800 chemical analyzer system (Siemens) on patient's sera.

### 2.4. Complete Blood Cell Count (CBC)

A CBC was performed using the Sysmex XE-2100 (Sysmex Corporation, Japan). Slides revised were prepared with SP-100 SYSMEX using a staining program was as follows: May-Grünwald (Bio Lyon, France) pure time: 2.5 min, MG dilute time: 3 min, Giemsa (Bio Lyon, France) time: 7 min, rinse 0 min, and drying time 5 min, as instructed by the supplier.

### 2.5. Statistics

Data were compared using the Mann-Whitney *U* test, Student's *t*-test, and Spearman's correlation coefficient when appropriate. The level of statistical significance was established at *P* < 0.05. Statistical analysis was conducted using SPSS 16.0 for Windows (SPSS Inc., Chicago, IL, USA).

## 3. Results

The description of the 100 patients and 61 healthy controls is summarized in Table 1. Of the SLE patients, 38% had lymphopenia (lymphocytes: <1200/uL), 13% had anemia (hemoglobin < 11 g/dL), 21% had neutropenia (neutrophils < 1500/uL), and 16% had thrombocytopenia (platelets < 150.000/uL). These disease manifestations and cell counts were at the time the blood sample was taken, and the patients were not subdivided by the number of cytopenic manifestations. None of these cytopenias were observed in the healthy control group. 

### 3.1. Neutrophil Analyses

**Figure 1:**
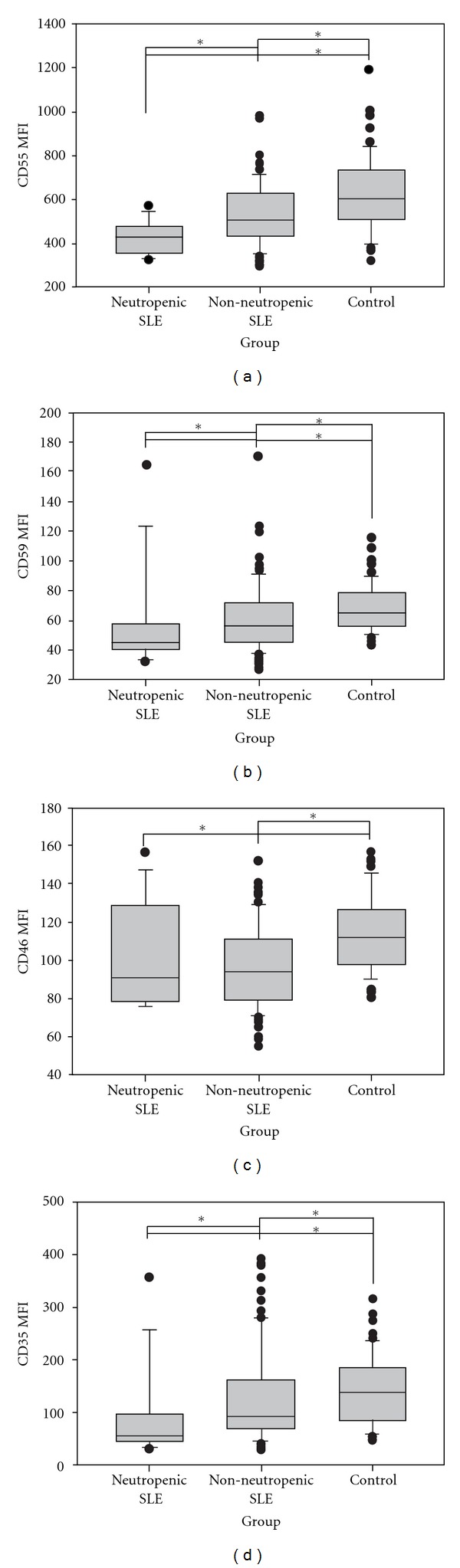
Creg surface expression of neutrophil cell. The figure displays mean fluorescence intensity (MFI) of CD55, CD59, CD46, and CD35 on gated neutrophil from SLE patients with neutropenia, without neutropenia and controls. Median and interquartile range from all subjects studied in each group were shown. *Significant statistical difference (*P* < 0.05).

In SLE patients, the MFIs of all Cregs on neutrophils (granulocytes) were significantly lower than those of healthy controls (Table 2). When comparing neutropenic (13/100) with non-neutropenic SLE patients, all Cregs, with the exception of CD46, were significantly decreased (Figure 1). 

There was a negative correlation between CD55 (*r* = −0.278, *P* = 0.019) and CD59 (*r* = −0.23, *P* = 0.048) expression on neutrophils and the SLEDAI; beside that, there was a positive correlation between CD55 (*r* = 0.237, *P* = 0.021) and CD35 (*r* = 0.334, *P* = 0.030) expression on neutrophils and C3 serum levels in SLE patients, and CD55 (*r* = 0.334, *P* = 0.001) with C4 level. 

When analyzing only neutropenic SLE patients, a positive correlation was shown between CD59 on neutrophils and C4 serum levels (*r* = 0.828, *P* = 0.006).

### 3.2. Lymphocyte Analyses

**Figure 2:**
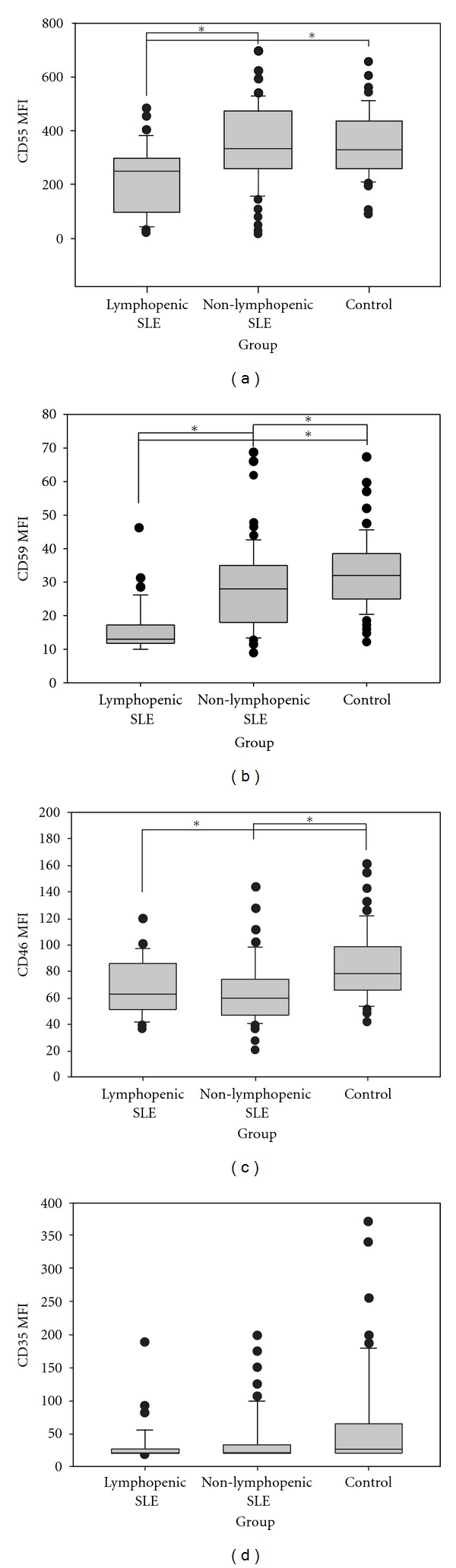
Creg surface expression of lymphocytes cell. The figure displays mean fluorescence intensity (MFI) of CD55, CD59, CD46, and CD35 on gated neutrophil from SLE patients with lymphopenia, without lymphopenia, and controls. Median and interquartile range from all subjects studied in each group were shown. *Significant statistical difference (*P* < 0.05).

In SLE patients, the MFIs of CD55, CD59, and CD46 on lymphocytes were significantly lower than those of healthy controls (Table 2). When comparing lymphopenic (38/100) with non-lymphopenic SLE patients, only CD55 and CD59 were significantly decreased (Figure 2). 

There was a positive correlation between CD55 (*r* = 0.231, *P* = 0.026) expression on lymphocytes and C3 serum levels in SLE patients, and no association with SLEDAI or SLICC.

### 3.3. Monocyte Analyses

In SLE patients, only the MFI of CD55 on monocytes was significantly lower than that of healthy controls (Table 2). There was no correlation between Creg expression on monocytes and C3 and C4 level or SLEDAI and SLICC in SLE patients.

### 3.4. Red Blood Cell Analyses

**Figure 3:**
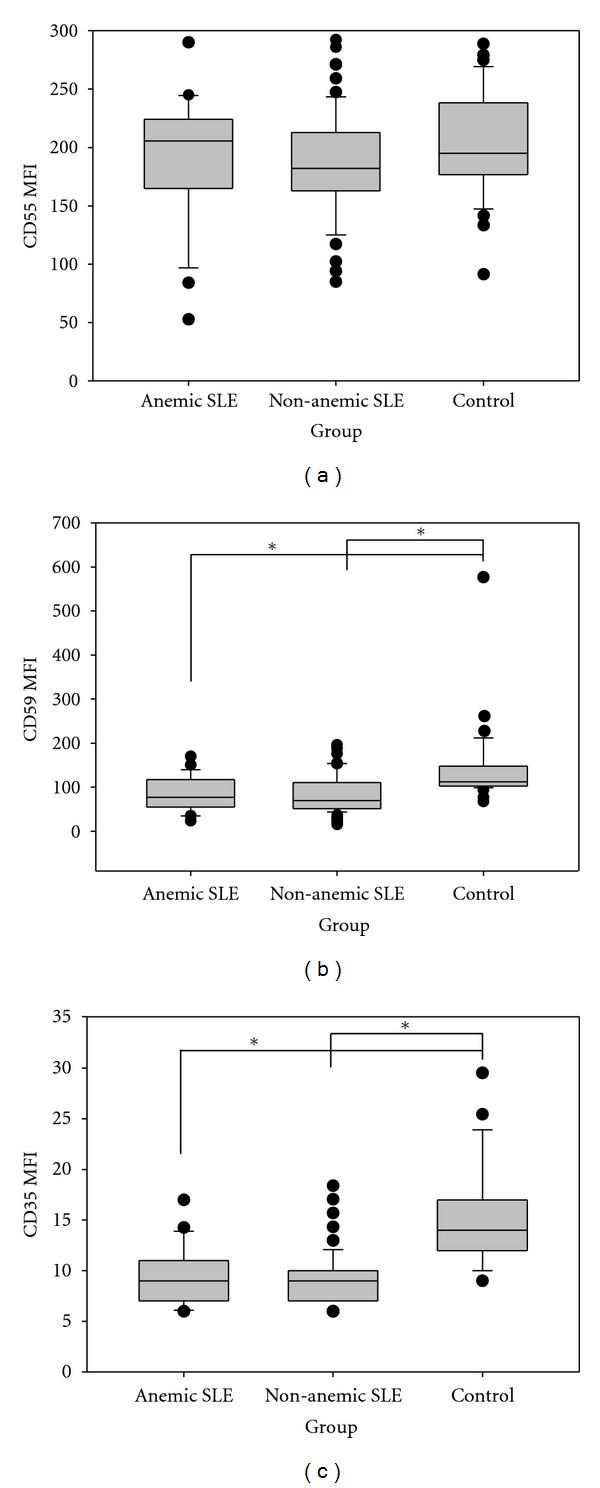
Creg surface expression of RBC. The figure displays mean fluorescence intensity (MFI) of CD55, CD59, CD46, and CD35 on gated RBC from SLE patients with anemia, without anemia, and controls. Median and interquartile range from all subjects studied in each group were shown. *Significant statistical difference (*P* < 0.05).

In SLE patients, the MFIs of CD59 and CD35 on RBC were significantly lower than those of healthy controls (Table 2). When comparing anemic (21/100) with nonanemic SLE patients, there were no MFI CD59 and CD35 statistic difference (Figure 3). 

There was a positive correlation between CD35 (*r* = 0.218, *P* = 0.049) expression on RBC and C4 serum levels in SLE patients and no association with SLEDAI or SLICC. When analyzed only anemic patients, this latter correlation was stronger (*r* = 0.526, *P* = 0.021). CD46 was not analyzed because it is not expressed on RBCs.

## 4. Discussion

Our study revealed significantly lower Creg expression on several blood cells from SLE patients when compared with healthy controls, more marked in cytopenic patients, and in many cases associated with higher disease activity and lower serum C3 and C4 levels. Although there are a few publications evaluating some of the Creg proteins in specific blood cells in SLE patients, our study is the first to encompass all the membrane-bound Cregs and all blood cells in a large sample of SLE patients. This allows a clear view of the expression profile of these proteins and their relations with decreased blood cell numbers and with disease activity. 

 We have previously reported a decreased expression of CD55 (but not of CD59) on neutrophils from SLE patients [21], and decreased CD35 expression on neutrophils has also been shown [16, 25]. In this study, beside confirming the decreased expression of CD55 and CD59, it was demonstrated that the higher the disease activity, the lower their expression on neutrophils. Furthermore, there might be a direct correlation between the lower CD55 and CD35 expression and activation of the classical complement pathway, as indicated by the lower C3 and C4 serum levels. These findings suggest that the decreased expression of Cregs may be due to their consumption trying to protect the cell against complement-mediated lysis, perhaps triggered by specific autoantibodies. 

On lymphocytes, the CD55, CD59, and CD46 MFI showed significant differences between SLE and controls. Lymphopenic patients presented the lower expression of these Cregs. Similarly to our results, Garcia-Valladares et al. [19] investigated the MFI of CD55 and CD59 in T and B lymphocytes from SLE patients with lymphopenia. Both T and B cells from lymphopenic patients showed decreased membrane expression of CD55 and CD59 when compared to controls. Tsunoda et al. [20] found that the proportion of CD59 on activated T CD8+ lymphocytes in SLE patients was significantly reduced compared to controls and that it could be correlated with disease activity and to be involved in the induced apoptosis of these cells. Our data showed that the decreased expression was unrelated to disease activity and accumulated damage using SLEDAI and SLICC, as has been reported [19, 21], but demonstrated that the lower the C3 level and consequently the greater complement activation, the lower the expression of CD55 on lymphocytes in these patients.

 The MFIs of CD59 and CD35 on RBCs from SLE patients were significantly reduced when compared to healthy controls, but this deficiency does not seem to be associated with anemia or autoimmune hemolytic anemia (AIHA), since the nonanemic and patients with no secondary AIHA also demonstrated reduced CD59 and CD35 MFI on their red cells. Our data about the decreased CD35 expression on RBC from SLE patients corroborate the findings of the literature [12–16]. Furthermore, we found that the low expression of CD35 in SLE patients was correlated with low C4 levels.

The diminished expression of CD59 on RBCs from SLE patients with secondary AIHA was previously reported by Richaud-Patin et al. [17]. However, in contrast with our results, SLE patients with no AIHA exhibited a normal expression of these molecules. It is important to mention that the number of patients evaluated in our study with and without AIHA was 28 and 72, respectively, which is much greater than that of the study of Richaud-Patin et al. 

We also observed a decreased CD35 and CD59 expression on RBCs from SLE patients with nephritis (*n* = 45) (*P* < 0.05, data not shown). This finding corroborates in part the findings of Arora et al. [18], who have demonstrated that, in 15 lupus nephritis patients, the expression of CD35 was significantly reduced compared to the expression on erythrocytes from normal individuals. On the other hand, these authors observed that CD55 and CD59 levels were highly elevated in RBCs, in contrast with our results. 

The cause of this generally decreased expression of Creg proteins in SLE blood cells is still unclear. Richaud-Patin et al. [17] have hypothesized that the diminished expression of CD55 and CD59 proteins on red cells might be due either to the impaired synthesis of the GPI (glycosylphosphatidylinositol) anchor or to the abnormal coupling of the protein to the membrane on red blood cell precursors. However, our findings do not support these hypotheses, since in that case the expression of Cregs would be uniformly reduced on all blood cells, while different patterns of diminished expression depending on each cell type were observed in our study.

A decline in CD35 expression at both mRNA transcript and protein level in SLE has been described, and it has been suggested to be acquired [26]. However, nothing is known about the factors involved in this downregulation of CD35 gene expression [27]. Lach-Trifilieff et al. [28] demonstrated that there is no lack of CD35 expression on young RBC (reticulocytes), in which CD35 is known to be low, and in most cases the low CD35 on RBC is due to an accelerated loss occurring in the circulation. Holme et al. showed that erythrocyte CD35 numbers are reduced during periods of increased disease activity and tend to return to normal during remission [29].

The fact that there was an association of decreased Creg expression with disease activity, low complement levels, and decreased peripheral blood cell numbers in our study indicates that the mechanism is related to the disease itself. The production of autoantibodies against specific cell self-antigens, Creg consumption, and complement-mediated lysis may be the most plausible explanation, as has also been partially suggested by other studies [5, 21, 30]. On the other hand, the use of immunosuppressive drugs may have influenced our results, being a limiting factor in our study and because of the nonhomogenous treatments and multiples therapies was limited to determine the clear association of a specific drugs with Creg decrease and/or cytopenia. We believe that the random inclusion of patients can reduce this influence if it really exists.

The decreased expression of the Cregs may also involve other functions of these proteins. For instance, CD59 has been implicated in the process of signal transduction and T-cell activation [31], and it has been reported that CD59 cross-linking induces internalization of this molecule and endocytosis of the lymphocyte membrane [32]. By another suggestion, it seems that the epitopes against which the monoclonal antibodies are directed somehow express themselves in a differential manner, depending on the cells' activation state [33].

In conclusion, it was evident that there are differences in the patterns of expression of Creg proteins on the peripheral blood cells from SLE patients, since the diminished MFI expressions of all Cregs proteins were found on neutrophils cells; CD55, CD59, and CD46 on lymphocytes; CD55 on monocytes; CD59 and CD35 on RBC. Moreover, these differences, even for the lower most part, seem to correlate with disease activity, complement activation, and blood cell cytopenias. The cause of the decreased expression on cell surface from SLE patients is not yet established, and the mechanisms by which cells are destroyed or sequestered remain rather obscure. We believe this is an adaptive phenomenon that happens due to a consumption of the Creg proteins when trying to prevent complement-mediated cell lysis. Moreover, the fact that each of these four hemopoietic lineages might show underexpression of Cregs independently from the others suggests the participation of different physiopathologic processes. Deeper understanding of these processes, and the role of Cregs, could be important for the development of novel therapies for the blood cell involvement in SLE and other autoimmune-mediated diseases.

## Figures and Tables

**Table 1:** Demographic, clinical, and laboratory features of SLE patients.

Patients' features	SLE (*n* = 100)	Healthy controls (*n* = 61)
Females (%)	93	67.2
Age (year) median (interquartile range)	42 (31–53)	45 (30–61)
SLEDAI^a^ median (interquartile range)	2 (0–5)	—
SLICC-DI^b^ median (interquartile range)	1 (0–2)	—
Malar rash (%)	58	—
Nephritis (%)	45	—
Arthritis (%)	67	—
AIHA^c^ (%)	28	—
RBC (×10^12^ cells/uL)	4.15 (0.55)^d^	4.4 (0.36)
Hemoglobin (g/dL)	12.0 (1.6)^d^	13.5 (1.2)
Platelets (×10^3^ cells/uL)	208 (65)^d^	228 (45)
Leucocytes (×10^3^ cells/uL)	5.43 (4.07–7.91)^e^	6.96 (6–8.59)
Lymphocytes (×10^3^ cells/uL)	1.32 (0.85–1.79)^e^	2.25 (1.75–2.85)
Neutrophils (×10^3^ cells/uL)	3.58 (2.22–5.29)^e^	3.77 (3.08–4.74)
Monocytes (×10^3^ cells/uL)	0.48 (0.37–0.68)^e^	0.58 (0.6–0.75)
Thrombocytopenia^∗^ (%)	16	0
Leukopenia^∗^ (%)	17	0
Lymphopenia^∗^ (%)	38	0
Neutropenia^∗^ (%)	13	0
Anemia^∗^ (%)	21	0
C4 level	25.4 (16.8)	—
C3 level	108.4 (28.1)	—

^a^SLEDAI: systemic lupus erythematosus disease activity index.

^b^SLICC-DI: systemic lupus international collaborating clinics damage index.

^c^AIHA: autoimmune hemolytic anemia (positive Coombs' test).

^d^Mean ± SD.

^e^Median (interquartile range).

^∗^Lymphopenia: <1200 lymphocytes/uL, neutropenia: <1500 neutrophils/uL, anemia: hemoglobin < 11 g/dL, and thrombocytopenia: platelets < 150.000 cells/uL.

**Table 2:** The mean of membrane fluorescence intensity (MFI) of CD55, CD59, CD46, and CD35 on the blood cells of SLE patients and controls.

Cell	Creg	SLE patient	Control	*P* ^b^
		MFI^a^	MFI^a^	
Neutrophils	CD55	515 ± 132	611 ± 168	0.001^∗^
	CD59	61 ± 24	68 ± 15	0.034^∗^
	CD35	88 (67–154)	138 (86–185)	0.007^∗^
	CD46	97 ± 21	113 ±19	<0.001^∗∗^

Lymphocytes	CD55	302 ±147	350 ± 121	0.041^∗^
	CD59	24 (13–31)	30 (25–38)	0.012^∗^
	CD35	23 (21–28)	28 (21–59)	0.053
	CD46	62 (49–77)	79 (65–97)	<0.001^∗∗∗∗^

Monocytes	CD55	953 ± 313	1057 ± 241	0.021^∗^
	CD59	23 (18–33)	22 (15.5–33)	0.422^∗^
	CD46	74 ± 21	78 ± 16	0.217
	CD35	122 (66.2–202)	138 (85–198)	0.296^∗∗^

RBC	CD55	188 ± 44	201 ± 43	0.153
	CD59	73 (53–110)	112 (102.5–148)	<0.001^∗∗^
	CD35	9.1 ± 2.5	15 ± 5.0	<0.001^∗∗^

^∗^Significant statistical difference (*P* < 0.05).

^∗∗^Significant statistical difference (*P* < 0.001).

^a^Media ± SD or median (25–75 interquartile range).

^b^Mann-Whitney *U* test or Student's *t*-test.
